# In Silico identification of a common mobile element insertion in exon 4 of *RP1*

**DOI:** 10.1038/s41598-021-92834-4

**Published:** 2021-06-28

**Authors:** Dongju Won, Joo-Yeon Hwang, Yeeun Shim, Suk Ho Byeon, Junwon Lee, Christopher Seungkyu Lee, Min Kim, Hyun Taek Lim, Jong Rak Choi, Seung-Tae Lee, Jinu Han

**Affiliations:** 1 Department of Laboratory Medicine, Yonsei University College of Medicine, 50 Yonsei-ro, Seodaemun-gu, Seoul, 03722 South Korea; 2 Division of Rare Diseases, Centers for Biomedical Sciences, Korea National Institute of Health, Korea Centers for Disease Control, Seoul, South Korea; 3 Division of Rare Disease Management, Bureau of Chronic Disease Prevention and Control, Korea Disease Control and Prevention Agency, Seoul, South Korea; 4 Department of Laboratory Medicine, Graduate School of Medical Science, Brain Korea 21 Project, Yonsei University College of Medicine KR, Seoul, South Korea; 5 Institute of Vision Research, Department of Ophthalmology, Shinchon Severance Hospital, Yonsei University College of Medicine, Seoul, South Korea; 6 Institute of Vision Research, Department of Ophthalmology, Gangnam Severance Hospital, Yonsei University College of Medicine, Eonjuro 211, Gangnamgu, Seoul, 06273 South Korea; 7 Department of Ophthalmology, Asan Medical Center, University of Ulsan College of Medicine, Seoul, South Korea

**Keywords:** Computational biology and bioinformatics, Genetics, Diseases

## Abstract

Mobile element insertions (MEIs) typically exceed the read lengths of short-read sequencing technologies and are therefore frequently missed. Recently, a founder *Alu* insertion in exon 4 of *RP1* has been detected in Japanese patients with macular dystrophy by PCR and gel electrophoresis. We aimed to develop a grep search program for the detection of the *Alu* insertion in exon 4 of *RP1* using unprocessed short reads. Among 494 unrelated Korean patients with inherited eye diseases, 273 patients with specific retinal phenotypes who were previously genotyped by targeted panel or whole exome sequencing were selected. Five probands had a single heterozygous truncating *RP1* variant, and one of their unaffected parents also carry this variant. To find a hidden genetic variant, whole genome sequencing was performed in two patients, and it revealed *Alu*Y c.4052_4053ins328/p.(Tyr1352Alafs*9) insertion in *RP1* exon 4. This *Alu*Y insertion was additionally identified in other 3 families, which was confirmed by PCR and gel electrophoresis. We developed simplified grep search program to detect this *Alu*Y insertion in *RP1* exon 4. The simple grep search revealed a median variant allele frequency of 0.282 (interquartile range, 0.232–0.383), with no false-positive results using 120 control samples. The MEI in *RP1* exon 4 was a common founder mutation in Korean, occurring in 1.8% of our cohort. The *RP1*-*Alu* grep program efficiently detected the *Alu*Y insertion, without the preprocessing of raw data or complex installation processes.

## Introduction

Inherited retinal diseases (IRD) are genetic eye diseases with high heterogeneity^1^. Next-generation sequencing (NGS) has improved the diagnostic rate of IRDs substantially. However, approximately 30–40% of patients do not achieve a definitive molecular diagnosis after whole-exome sequencing (WES) or even whole-genome sequencing (WGS)^2,3^. The disease-causing variants in these cases may be regulatory non-coding variants, deep intronic variants, mobile element insertions (MEIs), complex structural variants, or variants residing in repetitive low-complexity sequences, which are difficult to map or are easily missed in annotations of non-canonical transcripts. Improvements in bioinformatics and exome re-analysis method can increase the diagnostic rate for previously undiagnosed retinal diseases^2^.

Since the initial discovery of MEIs in corn by Barbara McClintock^4^, their crucial roles have been implicated in human diseases^5,6^. Several MEIs are associated with IRDs, such as retinitis pigmentosa (RP) or optic atrophy^7–11^. “Jumping gene” insertions caused by retrotransposons, such as long interspersed element 1 or short interspersed nuclear elements, can disrupt genes, leading to Mendelian disorders. These MEIs typically exceed read lengths for short-read sequencing technologies. Therefore, special bioinformatic approaches for transposable element identification, such as MELT and Mobster, have been developed^12,13^.

As transposable elements in the human genome account for approximately 45% of the total DNA content, it is difficult to determine whether certain MEIs are pathogenic. Recently, a founder *Alu* (a short interspersed nuclear element) insertion in exon 4 of *RP1* has been reported in Japanese patients with macular dystrophy^10,14,15^, as determined by optimized polymerase chain reaction (PCR)-based amplification with gel electrophoresis or Sanger sequencing. The genetic relatedness of Korean and Japanese populations suggests that founder *RP1*-*Alu* insertions may also be found in Koreans with macular dystrophy or cone-rod dystrophy (CRD). However, PCR and gel electrophoresis are time- and labor-intensive methods. Therefore, we developed a simple approach for detecting *Alu* insertions in *RP1* exon 4 from raw NGS data based on the known sequence of the mutant junction and applied the method to a Korean cohort with IRD with previously generated targeted panel NGS or WES data.

## Results

**Figure 1:**
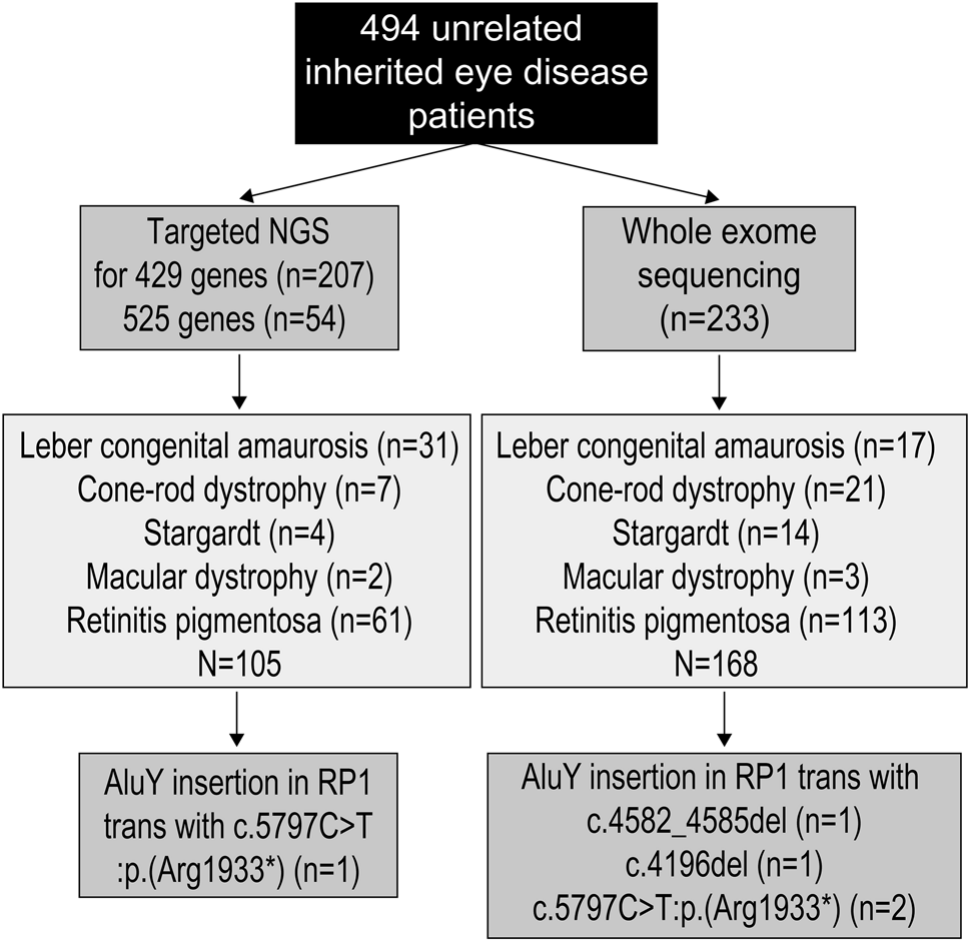
Inherited eye disease cohort. A total of 494 unrelated patients with inherited eye diseases underwent targeted panel next-generation sequencing or whole-exome sequencing. A subset of samples with Leber congenital amaurosis, cone-rod dystrophy, Stargardt disease, macular dystrophy, or retinitis pigmentosa underwent an additional *RP1*-Alu analysis. Among 273 patients, 1.8% patients had the *RP1*-Alu insertion.

**Figure 2:**
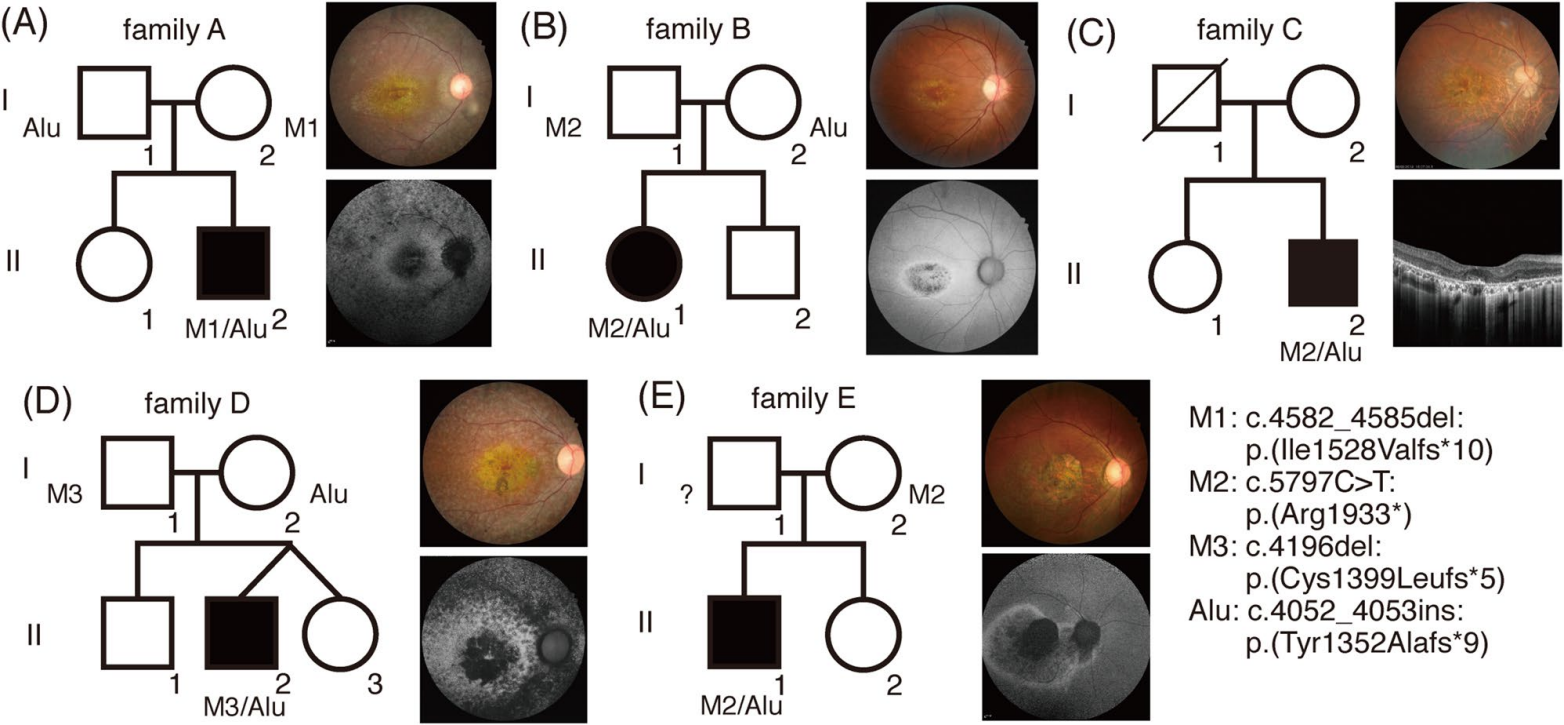
(**A**–**E**) Pedigree and retinal images of patients carrying the *RP1*-Alu insertion. Patients B, C, and E with c.5797C > T:p.(Arg1933*) had macular dystrophy without peripheral retinal degeneration. However, rod involvement around the retinal vascular arcade showed c.4582_4585del:p.(Ile1528Valfs*10) and c.4196del:p.(Cys1399Leufs*5).

**Figure 3:**
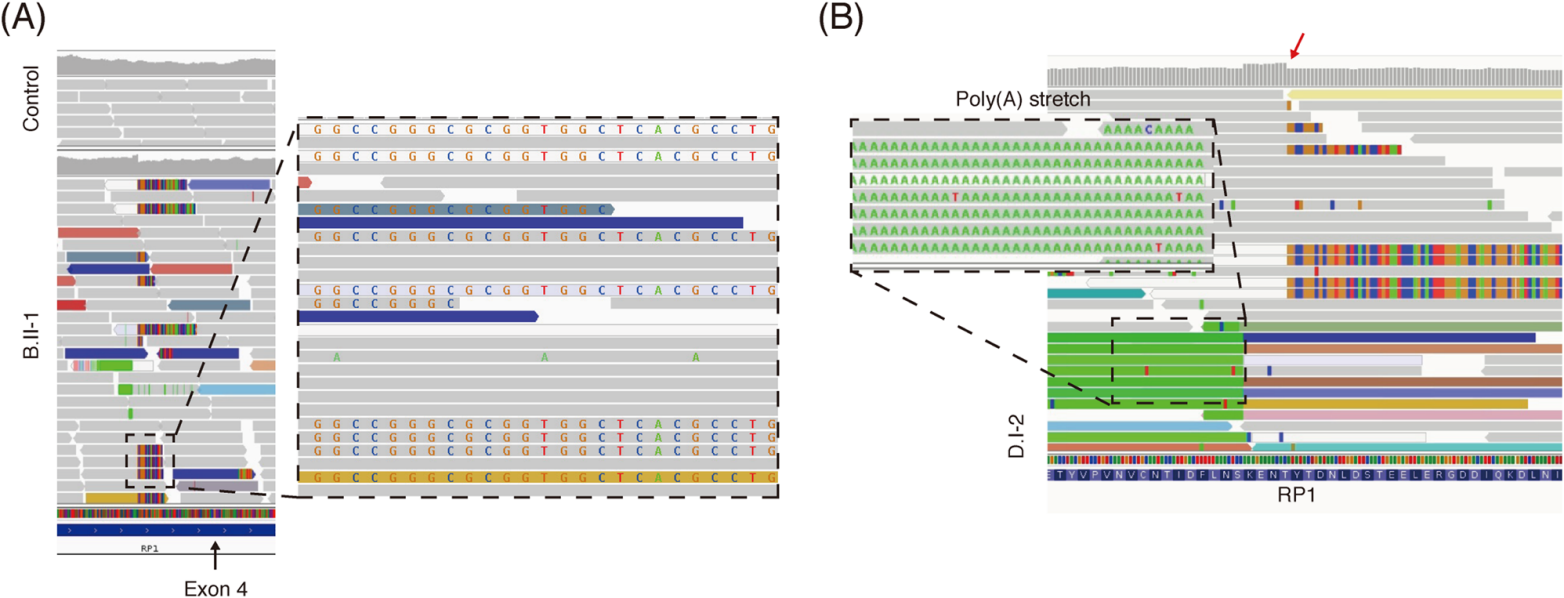
(**A**) Next-generation sequencing data from the Integrative Genomics Viewer at the Alu insertion junction from whole-genome sequencing of B.II-1 and one control sample. Integrative Genomics Viewer with “show soft-clipped bases” revealed multiple reads with aberrant alignments on the right side corresponding to an Alu Y insertion. (**B**) On the left side of aberrant soft-clipped bases from whole-genome sequences of D.I-2., Alu Y with the poly(A) tail was found. The space between the Alu insertion and poly(A) tail corresponds to target site duplication (sequence: AAAGAAAACAC). Coverage depth decreased sharply at the junction of the Alu insertion (red arrow).

At the time of the analysis, 233 patients with IRDs were sequenced and analyzed by WES (clinicaltrials.gov: NCT03613948) (Fig. 1), including 168 patients with Leber congenital amaurosis (LCA), CRD, Stargardt disease, macular dystrophy, and RP. We identified four unsolved cases with macular dystrophy or CRD carrying a single heterozygous truncating mutation in *RP1* (NM_006269.1) based on a WES analysis (Fig. 2 and Figure S1). However, autosomal dominant inheritance was unlikely because an unaffected parent also had this variant and the minor allele frequency (MAF) was high. Additionally, the variants were located in a region with autosomal recessive inheritance^16^. Trio-based WGS of family D and proband-only WGS of one proband (family B) revealed no *RP1* copy number variants or structural variants. The *RP1* genomic region showed a more complex event leading to incorrect variant calling. A WGS analysis of one patient (family B) revealed the c.4052_4053insGGCCGGGCGCGGTGGCTCACGCCTGTAATCCCAGCACTTTGGG: p.(Tyr1352Alafs*9) variant along with c.5797C > T:p.(R1933*), with a low variant allele frequency (VAF) (19.6%, allele depth = 41:10, REF:ALT) and VQSRTrancheINDEL 99.95–100.00. Genome Analysis Toolkit uses a machine learning model to differentiate true variants from false positives. A VQSRTrancheINDEL of ≥ 99.00 corresponds to tranches with more false positives. Therefore, VQSRTrancheINDEL 99.95–100.00 indicates a high probability of false positives. However, the soft clipped part of reads (i.e., the longest 122 bp) and opposite side of the poly-A tail in the WGS analysis of B.II-1 revealed an insertion of an *Alu*Y retrotransposon at chr8:55,540,494 (hg19) (Fig. 3A,B). WES or WGS analyses of the four probands (A–D) and the mother of unaffected patient D yielded similar results. Accordingly, we re-examined targeted NGS data for 105 patients with LCA, CRD, Stargardt disease, macular dystrophy, or RP (Fig. 1). We identified one additional patient (family E) with macular dystrophy previously thought to be unsolved because he harbored only one heterozygous nonsense c.5797C > T variant in *RP1* (Fig. 2E). Likewise, abnormal reads with low VAFs were suspected between c.4052 and c.4053 in *RP1,* as determined using Integrative Genomics Viewer, but no variants were called at the position.

To better detect *Alu* insertions at this location in *RP1* in patients with macular dystrophy or CRD, we designed a simple grep search program including the reference sequence (13 bp) and *Alu*Y sequence (13 bp) at the junction. We identified an *Alu* insertion in *RP1* exon 4 in unsolved patients with the disease-causing variant p.(Arg1933*) in families B, C, and E, p.(Ile1528Valfs*10) in family A, and p.(Cys1399Leufs*5) in family D. Interestingly, macular dystrophy without peripheral retinal dystrophy was observed with c.5797C > T:p.(Arg1933*) and early-onset CRD was observed with c.4582_4585del:p.(Ile1528Valfs*10) (family A.II-2) and c.4196del:p.(Cys1399Leufs*5) (family D.II-2). The latter two patients had childhood-onset nystagmus and were legally blind at the age of 20 years (Table 1).

**Table 1:** Summary of demographic factors, visual acuity, and genetic data for the *RP1*-Alu insertion case series.

Sample_name	Sex	Current Age (years)	Age of Onset (years)	BCVA		Refractive Error (SE)	*RP1* disease-causing variants (NM_006269.1)	Fundus autofluorescence	Clinical diagnosis
				OD	OS				
A.II-2	M	18	10	20/250	20/800	OD: -2.75 OS: -2.25	c.4582_4585del:p.(Ile1528Valfs*10) c.4052_4053ins328:p.(Tyr1352Alafs*9)	Central hypofluorescence with peripheral pathy-like hypofluorescence	Cone-rod dystrophy
B.II-1	F	30	11	20/250	20/200	OD: -5.50 OS: -5.00	c.5797C > T:p.(Arg1933*) c.4052_4053ins328:p.(Tyr1352Alafs*9)	Central hypofluorescence surrounded by a hyper fluorescent halo	Macular dystrophy
C.II-2	M	42	18	20/100	20/50	OD: -7.25 OS: -6.25	c.5797C > T:p.(Arg1933*) c.4052_4053ins328:p.(Tyr1352Alafs*9)	NA	Macular dystrophy
D.II-2	M	20	7	HM	CF	OD: -2.50 OS: -1.75	c.4196delG:p.(Cys1399Leufs*5) c.4052_4053ins328:p.(Tyr1352Alafs*9)	Central hypofluorescence with peripheral pathy-like hypofluorescence	Cone-rod dystrophy
E.II-1	M	38	20	20/200	20/100	OD: -7.50 OS: -7.25	c.5797C > T:p.(Arg1933*) c.4052_4053ins328:p.(Tyr1352Alafs*9)	Central hypofluorescence surrounded by a hyper fluorescence halo that extends nasally of the optic disc	Macular dystrophy

*BCVA* best-corrected visual acuity, *CF* count finger, *HM* hand motion, *NA* not available, *OD* oculus dexter, *OS* oculus sinister, *SE* spherical equivalent.

*Alu* insertions between c.4052 and c.4053 in *RP1* were suspected for the five patients described above. We confirmed the *Alu* insertion in exon 4 of *RP1* by PCR and gel electrophoresis using samples from four patients and their available parents (Figure S2); patient D was excluded owing to the lack of residual sample. An approximately 300-bp insertion was identified in the probands (family A–C and E); in the probands of families A and B, the insertion originated from the father and mother, respectively. The mother of the proband in family E had no *RP1* insertion; thus, the insertion likely originated from the father. Sanger sequencing of *RP1* of the parents in families A and B and WGS of the parents in family D revealed that the insertion and another truncating variant in *RP1* in families A, B, and D were located in trans. Sanger sequencing revealed that *RP1* c.5797C > T:p.(Arg1933*) in the proband of family E originated from the mother, indirectly confirming that the variant and *Alu* insertion in the proband of family E were in trans. The *Alu* sequence was determined by Sanger sequencing of a purified ~ 672 bp band in gel electrophoresis. Except for the poly(A) tail, 5 of 282 bases in *Alu* differed from the previously reported *Alu* Y reference (Figure S3)^17^. Interestingly, some bases preceding the *Alu* insertion were detected behind the poly(A) of *Alu* Y in duplicate. These two direct repeats were likely introduced during the *Alu* insertion. The *Alu* sequence reported in the Japanese population has not been reported and thus it was not possible to confirm that the same element was present. However, the high prevalence in cases in both Korea and Japan and the identical position strongly suggest that the event was a common founder effect. The predictive pathogenicity and population frequency of the variants are summarized in Table S1.

### Validation of the grep search

Using the bash grep command, we found a median VAF of 0.282 (interquartile range, IQR, 0.231–0.383) in nine sets of sequencing data from six patients (five probands and the mother of proband D) for the heterozygous *RP1*-*Alu* insertion (Table S2). The median VAF was 0.229 in targeted panel sequencing data (n = 2), 0.261 (IQR, 0.201–0.333) in WES (n = 4), and 0.447 (IQR, 0.382–0.452) in WGS (n = 3) (Table S2). To validate the grep search method, we applied it to targeted panel sequencing (n = 20), WES (n = 80) and WGS (n = 20) samples with other diseases, such as *FRMD7*-related infantile nystagmus, congenital cataract, or inherited optic neuropathy. No mutant reads were detected in these control samples.

### Comparison with other mobile element detection tools

We used the MELT, Mobster, and SCRAMble tools to compare the efficacy and runtime for MEI detection in *RP1* exon 4^12,13,18^. The *RP1*-*Alu* was not called in two targeted NGS samples using the MELT algorithm and in one WES sample (Patient C.II-2) using the Mobster and SCRAMble algorithms (Table S3).

**Figure 4:**
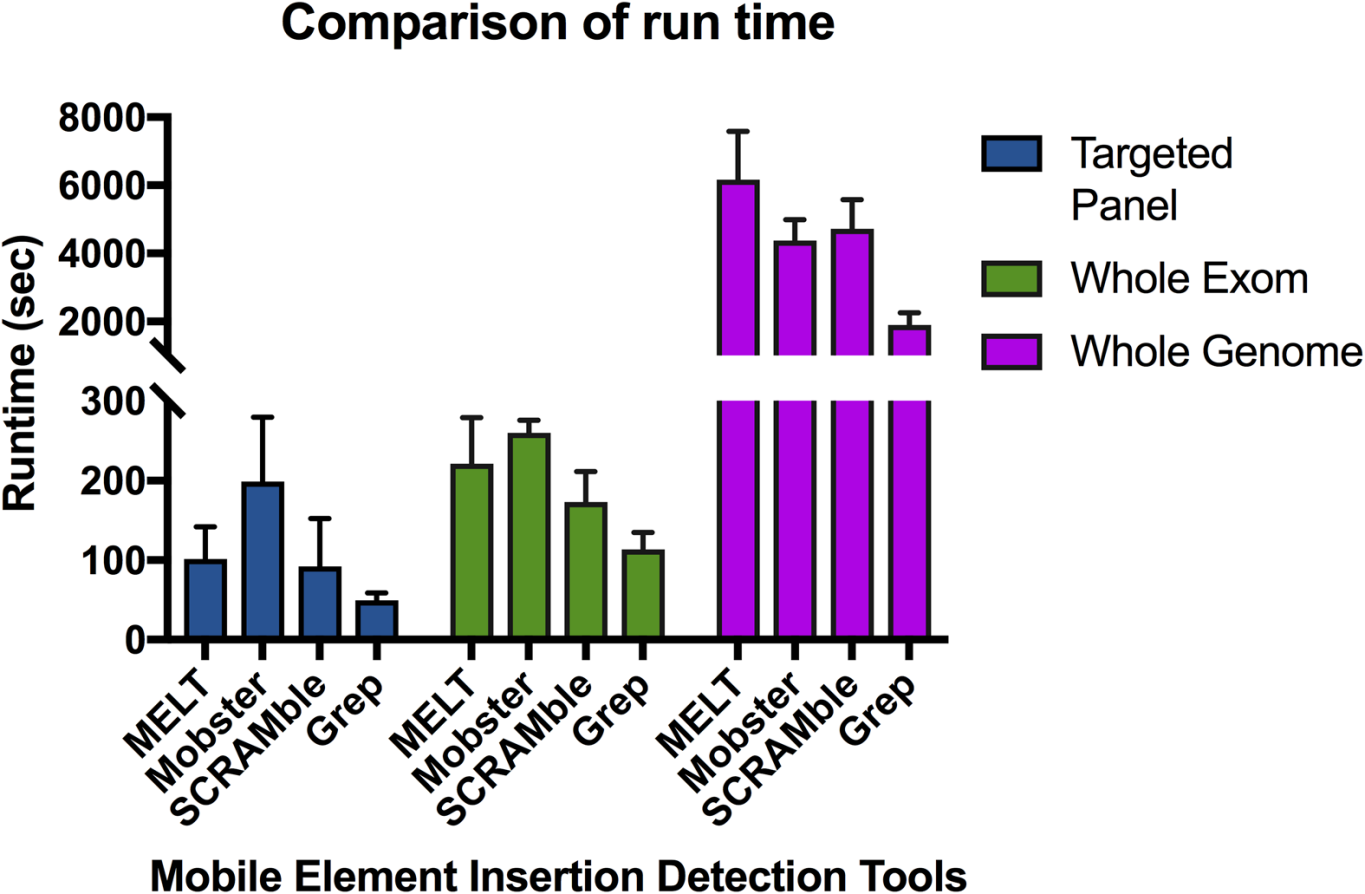
Runtimes of each tool for the detection of mobile element insertion. All tools were run using dual Xeon E5-2643V4 (12-core, 24 threads) and 64 GB of RAM.

Computation time is a limiting factor when running MEIs detection tools using large datasets. For targeted panel data, the median runtimes were 101.5 s (IQR, 73–130 s) for MELT, 199 s (IQR, 142–256 s) for Mobster, 92.5 s (IQR 50–135 s) in SCRAMble, and 49.5 s (IQR, 43–56 s) for the grep program. For WES data, the median runtimes were 240 s (IQR, 179–263.5 s) for MELT, 273 s (IQR, 256.5–312.5 s) for Mobster, 172.5 s (IQR, 140–206.5 s) for SCRAMble, and 117.5 s (IQR, 100.5–126.5) for the grep program. For WGS data, the median runtimes were 91 min (IQR, 87.1–130.1 min) for MELT, 70.6 min (IQR, 64.4–84.1 min) for Mobster, 71.1 min (IQR, 69.7–95.2 min) for SCRAMble, and 29.0 min (IQR, 27.7–38.5 min) for the grep program (Fig. 4). The runtimes did not account for the pre-processing, filtering, and annotation of MEIs.

## Discussion

*RP1* is located on chromosome 8 and comprises 4 exons (3 coding) and 2156 amino acids. Most of reported disease-causing variants are clustered in the largest and terminal exon 4, and *RP1* disease-causing variants show autosomal dominant or recessive inheritance patterns depending on the type and position of variants^16^. We have found 5 unsolved cases with a single disease-causing variant in the *RP1* region with autosomal recessive inheritance based on NGS data. The c.4052_4053ins328 *Alu* element insertion in *RP1* seems to be the second variant in East Asian population.

Several MEIs have been implicated in IRDs, including RP, choroideremia, or autosomal dominant optic atrophy^7,11,19,20^. The *Alu*Yb8 insertion in *MAK* is a founder mutation in the Jewish population^8^, and a *BBS1* SVA F retrotransposon insertion is a frequent cause of Bardet-Biedl syndrome in Europeans^11^. Furthermore, recent studies have identified MEIs as causative mutations in 0.04–0.15% of cases^18,21^. The *MAK*-*Alu* grep program is an efficient tool for the detection of founder MEIs in the Jewish population^22,23^. Studies aimed at detecting pathogenic MEIs in Asian populations are relatively limited, despite the potential for population-specific founder MEIs. Recently, a founder *Alu* insertion in exon 4 of *RP1* has been reported with autosomal recessive inheritance in Japanese patients with macular dystrophy^10,14,15,24^. Therefore, the founder MEI found in the Japanese population should be also investigated in the Korean population.

MEIs can often be missed by NGS methods due to PCR amplification and targeted capture in both targeted panel and WES data. PCR and gel electrophoresis have been used to identify the *Alu* in exon 4 of *RP1* in cases with a heterozygous, disease-causing variant in *RP1* by targeted panel sequencing^15^. However, this approach is time-consuming, expensive, and laborious. Therefore, we created a grep search program to detect the Alu in exon 4 of *RP1* with previously generated raw NGS data, without requiring further experiments. By incorporating the simplified grep program in our clinical diagnostic pipeline, we detected MEI in *RP1*, which can provide a definitive molecular diagnosis that is typically missed by short-read sequencing. In our cohort with compatible phenotypes (n = 273), MEI was detected in 1.8% of patients, consistent with the frequencies reported in previous studies^24^. Numa et al. suggested that tier-based approach is more efficient to detect pathogenic variants in Japanese RP as following orders: (1) Sanger sequencing of two major mutations in *EYS*, (2) targeted sequencing of all *EYS* coding exons, (3) WGS, and (4) Sanger sequencing of the *Alu* element in *RP1*^24^. However, our grep program detected the *RP1-Alu* insertion efficiently without further experiments or sequencing and yielded no false-positives. Because this simple bash or R script does not require the preprocessing of raw data or a complex installation process, it can be easily integrated in any NGS pipeline for the analysis of East Asian patients with IRD.

*RP1*-*Alu* is absent from publicly available resources, such as the 2,504 controls in the 1,000 Genomes Project and 14,891 controls in gnomAD SV 2.1. However, the c.5797C > T:p.(Arg1933*) variant is relatively common in Korean and Japanese individuals. The MAF was 0.0060 in gnomAD Korean and 0.0021 in gnomAD East Asian. The MAF of c.5797C > T is also high (0.0076) in the Korean Reference Genome Database (1722 samples, http://coda.nih.go.kr/coda/KRGDB/index.jsp). This variant does not cause retinal dystrophy in either homozygous or heterozygous individuals^10^. Interestingly, c.5797C > T with the *Alu* insertion causes macular dystrophy without peripheral retinal dystrophy.

However, the *Alu* insertion in trans with more proximal frameshift mutations (c.4196del or c.4582_4585del) causes childhood-onset nystagmus and severe macular dystrophy with rod involvement, consistent with early-onset CRD. It occurs during childhood, with the first symptoms recognized in the first decade^25^. When compared with that in LCA, visual function in early-onset CRD is slightly better, but progressive loss of retinal function leads to blindness in the second to third decade of life. We found that the *RP1*-*Alu* variant along with other frameshift mutations can cause childhood-onset retinal dystrophy with nystagmus, mimicking LCA or Stargardt disease. As *RP1* mutations cause CRD, RP, or macular dystrophy in either autosomal recessive or dominant states depending on the mutation location and type^26^, careful evaluations of the family history and the locations of variants in *RP1* are important, particularly when a single heterozygous disease-causing *RP1* variant is found and the family history does not indicate autosomal dominant inheritance.

MEIs can be detected using the MELT or Mobster algorithm based on discordant read pairs and clipped reads in combination with consensus sequences of known mobile elements^12,13^. Additionally, SCRAMble shows relatively high sensitivity for the detection of MEIs occurring within a targeted capture region^18^. These tools show reduced sensitivity for target enrichment sequencing relative to PCR-free genome sequencing because discordant read pairs can exist outside of target regions. Indeed, *RP1*-*Alu* was not detected in two targeted NGS samples using the MELT algorithm and in one WES sample using both Mobster and SCRAMble. Furthermore, our grep has various practical advantages over other algorithms, including the reduced computational time, no need for complex installation processes or preprocessing steps.

Despite these advantages, it should be emphasized that our *RP1*-*Alu* grep program is only useful to detect the founder MEI in exon 4 of *RP1*. Although no common variants have been reported within 13 bp upstream of the *Alu* insertion in gnomAD v2.1.1, a rare variant was found in gnomAD v3.1 (hg38: 8–54,627,925-A-G: MAF = 1/152,184) 10 bp upstream of the *Alu* insertion site. To allow one mismatch within junction of the *Alu* insertion, R agrep program will yield positive results in such cases. We were also unable to confirm the validity of the method in patients with a homozygous *RP1*-*Alu* insertion or in other populations.

In conclusion, our results showed that the *RP1*-*Alu* insertion is common in Koreans with IRD, occurring in 1.8% of patients with IRD. *RP1*-*Alu* grep detected this common MEIs with no false-positive results. These findings provide a basis for further studies of the founder *RP1*-*Alu* insertion in pre-existing NGS data in East Asian patients with unsolved IRD. We also determined the full sequence of the inserted *Alu* Y. In unsolved early-onset CRD or macular dystrophy, *RP1*-*Alu* should be investigated using short-read sequencing data in East Asians.

## Methods

### Patient cohort and *Alu* detection process

The study protocol adhered to the tenets of the Declaration of Helsinki and was approved by the Institutional Review Boards of Yonsei University College of Medicine, Gangnam Severance Hospital (3–2020-0330). All probands were unrelated. Patients with clinical information were recruited and clinically examined at Severance Hospital, Yonsei University College of Medicine. Informed consent was obtained from all subjects or, for subjects under 18 years of age, from a parent or legal guardian; informed consent included consent for the publication of identifying information/images. Blood samples were collected for DNA extraction; 494 unrelated patients with inherited eye diseases, including *FRMD7*-related infantile nystagmus, congenital cataract, Stickler syndrome, familial exudative vitreoretinopathy, inherited optic atrophy, PR, LCA, CRD, and macular dystrophy, were identified. In total, 261 patients were evaluated by targeted panel NGS and 233 patients were evaluated by WES using xGen Exome Research Panel v1 (Integrated DNA Technologies, Coralville, IA, USA) and Twist Human Comprehensive Exome (Twist Bioscience, San Francisco, CA, USA). Proband-only WGS or trio WGS was additionally performed for 16 unresolved cases after targeted panel NGS or WES. Sequencing and bioinformatic analyses were performed as described previously and are summarized briefly in the Supplement methods^27,28^. Probands with LCA, Stargardt disease, CRD, macular dystrophy, and RP were screened. We evaluated unsolved patients with only one disease-causing variant in *RP1* for selected probands and implemented a newly developed grep search program with FASTQ files to detect the *Alu* insertion in exon 4 of *RP1*. We additionally tested the program using control samples. Suspected *Alu* insertions in *RP1* were confirmed by PCR and electrophoresis.

### Grep program to detect *RP1*-*Alu*

The Linux grep command was used to search FASTQ files for the 5′ junction between the reference sequence of exon 4 and the beginning of the *Alu* insertion in *RP1*. Most FASTQ files without the insertion returned a count of "0," though in rare cases a false-positive read count of 1 or 2 was detected in wild-type samples depending on the coverage depth and sequencing method. The variant allele frequency (VAF) was calculated as mutant read count/(wildtype read count + mutant read count). The program returns “No *Alu*Y insertion: VAF < 0.1,” “*Alu*Y insertion suspected: 0.1 ≤ VAF < 0.3,” or “*Alu*Y insertion detected: VAF ≥ 0.3.” The grep search program is described in detail in the Supplementary methods.

### The comparison of Mobile Element Insertion (MEI) detection callers

We compared efficacy and runtimes with MELT (v2.2.2, https://melt.igs.umaryland.edu/)^12^, Mobster (v0.2.4.1, https://github.com/jyhehir/mobster)^13^, and SCRAMble (v1.0.1, https://github.com/GeneDx/scramble)^18^, for NGS sequencing data from targeted panel sequencing (n = 2), WES (n = 4) and WGS (n = 3) samples. All tests were done with default setting, and -exome TRUE parameter was used only for targeted panel sequencing and exome sequencing in MELT algorithm. The real wall clock time in time command was used to check the runtimes. All tools were run using dual Xeon E5-2643V4 (12-core, 24 threads) and 64 GB of RAM.

## Supplementary Information

Supplementary Information.

## Data Availability

Data supporting the findings of this manuscript are available from the corresponding author upon reasonable request.

## Code availability

RP1-Alu detection tools are available on GitHub for non-commercial use (https://github.com/jin0008/RP1_aluinsertion).
