# Supplementary material for: In Silico identification of a common mobile element insertion in exon 4 of RP1
Source: Sci Rep. 2021 Jun 28;11:13381. doi: 10.1038/s41598-021-92834-4 (PMC8238996; doi:10.1038/s41598-021-92834-4)
Supplement: Supplementary file 1 — Supplementary Information. [file 41598_2021_92834_MOESM1_ESM.docx]

**Supplementary Information**

**In Silico Identification of Common Mobile Element Insertion in the Exon 4 of *RP1***

Dongju Won,^1,*^ Joo-Yeon Hwang,^2,3,*^ Yeeun Shim,^4^ Suk Ho Byeon,^4,5^ Junwon Lee,^6^ Christopher Seungkyu Lee,^5^ Min Kim,^6^ Hyun Taek Lim,^7^ Jong Rak Choi,^1^ Seung-Tae Lee,^1,*^ Jinu Han,^6,*^

^1^Department of Laboratory Medicine, Yonsei University College of Medicine, Seoul, South Korea

^2^Division of Rare Diseases, Centers for Biomedical Sciences, Korea National Institute of Health, Korea Centers for Disease Control, South Korea

^3^Division of Rare Disease Management, Bureau of Chronic Disease Prevention and Control, Korea Disease Control and Prevention Agency, South Korea

^4^Brain Korea 21 Project for Medical Science, Yonsei University College of Medicine, Seoul, South Korea

^5^Institute of Vision Research, Department of Ophthalmology, Shinchon Severance Hospital, Yonsei University College of Medicine, Seoul, South Korea

^6^Institute of Vision Research, Department of Ophthalmology, Gangnam Severance Hospital, Yonsei University College of Medicine, Seoul, South Korea

^7^Department of Ophthalmology, Asan Medical Center, University of Ulsan College of Medicine, Seoul, South Korea

**Supplementary methods**

**Targeted Panel Sequencing and Whole Exome Sequencing (WES)**

For the customized NGS panel, we selected 429 genes (n=207) and 525 genes (n=54) known to cause inherited eye diseases, based on our assessment of literature reviews, the RetNet database (https://sph.uth.edu/Retnet/), and the Online Mendelian Inheritance in Man database (https://www.ncbi.nlm.nih.gov/omim); we also included deep intronic or regulatory regions known to cause inherited eye diseases. Target enrichment was performed with custom-designed RNA oligonucleotide probes and target enrichment kit (Celemics, Seoul, South Korea). Whole exome sequencing was performed using xGen Exome Research Panel v1 (Integrated DNA Technologies, Coralville, Iowa, USA) or Twist Comprehensive Exome Kit (Twist Bioscience, San Francisco, CA, USA). Briefly, pooled libraries were sequenced using a NextSeq 550 sequencer (Illumina, San Diego, CA, USA) and the NextSeq Reagent Kit, version 2 (300 cycles) for the targeted panel sequencing and pooled libraries were sequenced using a NovaSeq 6000 sequencer (Illumina, San Diego, CA, USA). For each sample, quality metrics were calculated using the FastQC software and TEQC package. Sequences were aligned to the hg19 reference genome using BWA-aln. Single nucleotide variants and small insertions or deletions were called and crosschecked using GATK version 3.8.0 with Haplotypecaller and VarScan version 2.4.0. Each variant suspected to be pathogenic, likely pathogenic, or variant of uncertain significance (VUS) was confirmed by visual inspection of the bam file using the Integrated Genomics Viewer 2.3 software. Split-read based detection of large structural variations was conducted using Pindel and Manta. Read-depth based detection of copy number variation (CNV) was conducted using ExomeDepth version 1.1.10., followed by visualization using a base-level read depth normalization algorithm designed by the authors. CopywriteR version 2.9.0 was used with a 1-Mb window option for off-target analysis and whole chromosomal CNV detection.

**Variant Filtering and Classification**

Databases used for analysis and variant annotation include the Online Mendelian Inheritance in Man (OMIM), Human Gene Mutation Database, ClinVar, Single Nucleotide Polymorphism database (dbSNP), 1000 Genome, Exome Aggregation Consortium (ExAC), Exome Sequencing Project, and Korean Reference Genome Database, with a minor allele frequency cut-off of 0.5%. The pathogenicity of missense variants was predicted using 5 *in silico* prediction algorithms, including SIFT, PolyPhen2, FATHMM, and CADD. Splice site analysis was performed using the MaxEntScan, NNSPLICE (Neural Network Splice Prediction), Human Splice Finder, GeneSplicer, and SpliceFinder-like algorithms implemented in the Alamut Visual software (Interactive Biosoftware, Rouen, France). The interpretation of variants followed the 5-tier classification system recommended by the American College of Medical Genetics and Genomics and the Association for Molecular Pathology.

**PCR validation of RP1-*Alu* insertion**

PCR and gel electrophoresis were performed to confirm the *Alu* insertion in exon 4 of RP1 in patients with “*Alu* Y insertion suspected” or “*Alu*Y insertion detected” by RP1-*Alu* detection tools. In-house primers were selected after testing several primers (forward: 5′-AAGGCTTGTGCTCAAAAGGA-3′, reverse: 5′-CACTTAGGCAAAGGCCACAG-3′). PCR was performed in a 50 μL volume containing 50 ng of genomic DNA, 10× EF-Taq buffer, 10 mM dNTPs, 10 μM each primer, and 1.25 units (2.5 U/μL) of Solg EF-Taq DNA polymerase (Solgent, Daejeon, South Korea). PCR products were quality-checked by agarose gel electrophoresis. To determine the size of the inserted *Alu*, the *Alu*-inserted DNA fragment was extracted from the gel using a QIAquick Gel Extraction Kit (Qiagen, Hilden, Germany), and Sanger sequencing was performed. The extracted DNA fragment was purified using MG Exo-AP PCR Clean-Up Mix (MGmed, Seoul, South Korea) and sequencing was performed in a 5.5 μL sample containing 1 µL of Exo-AP product, 1 μL of primer (1 µM), 0.3 μL of BigDye Terminator v3.1, and 0.5 μL of the provided buffer (Applied Biosystems, Foster City, CA, USA). The sequencing product was cleaned by ethanol precipitation and sequenced on an ABI 3730xl (Applied Biosystems).

**Bash script to detect RP1 *Alu* insertion**

In a *RP1*-*Alu* containing sample, the program returns a positive value depending on the coverage depth in that area (typically 21-55 reads but as low as 13). Most files without the insertion return a count of “0” though rarely a false-positive read count of 1 or 2 can be detected in minority of wildtype samples. The requirement to exactly match the extended reference sequence, as currently implanted, has the disadvantage that, theoretically, a second-site SNP near the junction could prevent matching the full “reference” sequence; this false-negative result was not observed in the current data sets and is probably rare in this haplotype.

**<multi-thread mode> # required to install GNU parallel**

echo ""

echo "RP1-Alu grep search start"

echo ""

for sample_id in *_R1.fastq.gz;

do SAMPLE=${sample_id%%_R1.fastq.gz}; \

find $SAMPLE*.fastq.gz -type f | parallel -j+1 zgrep -c -e ACCGCGCCCGGCCGTGTTTTCTTTGG -e CCAAAGAAAACACGGCCGGGCGCGGT \

| awk '{sum += $1} END {print sum}' > $SAMPLE.mutantcount;

find $SAMPLE*.fastq.gz -type f | parallel -j+1 zgrep -c -e GTTATCAGTATATGTGTTTTCTTTGG -e CCAAAGAAAACACATATACTGATAAC \

| awk '{sum += $1} END {print sum}' > $SAMPLE.wildtypecount;

paste -d+ $SAMPLE.mutantcount $SAMPLE.wildtypecount | bc > $SAMPLE.depth;

paste $SAMPLE.mutantcount $SAMPLE.depth | awk '{print($1/$2)}' > $SAMPLE.VAF;

value=( $(<$SAMPLE.VAF));

X=0.1;

Y=0.3;

if [ $(echo " $value < $X" | bc) -eq 1 ]; then

echo "VAF < 0.1 : No AluY insertion was found in exon 4 of RP1 at 8:55540494 position (hg19)" > $SAMPLE.RP1_Alu.txt

elif [ $(echo " $value >= $Y" | bc) -eq 1 ]; then

echo "0.1 <= VAF < 0.3 : AluY insertion was suspected in exon 4 of RP1 at 8:55540494 position (hg19). Please recheck by visualizing AluY sequence at 8:55540494 position (hg19)" > $SAMPLE.RP1_Alu.txt

else

echo "VAF >= 0.3 : AluY insertion was detected in exon 4 of RP1 at 8:55540494 position (hg19)" > $SAMPLE.RP1_Alu.txt

fi

done;

**R script to detect RP1 *Alu* insertion**

In a *RP1*-*Alu* containing sample, the program returns a positive value depending on the coverage depth in that area (typically 21-55 reads but as low as 13). Most files without the insertion return a count of “0” though rarely a false-positive read count of 1 or 2 can be detected in minority of wildtype samples. This R script allow a second-site SNP near the junction (13 bp) using agrep search.

**Fastqsplit.R**

dir.create('split')

TotalThread=as.numeric ( Sys.getenv ('TotalThread') )

fqpattern='.fastq.gz$'

fqs=list.files(pattern=fqpattern)

samples = levels(as.factor(gsub('.', '', gsub('_$', '', gsub('R1.fastq.gz$|R2.fastq.gz$', '', fqs)), fixed=T)))

writeLines (samples, 'samples.txt')

for (i in 1:length(samples)){

Sample=samples[i]

samplefq=fqs[grepl(Sample,fqs)]

fq1=samplefq[grepl('R1.fastq.gz',samplefq)][1]

fq2=samplefq[grepl('R2.fastq.gz',samplefq)][1]

system (paste0 ( 'zcat ', fq1, ' ', fq2, ' | sed -n \'2~4p\' > ', Sample, '.seqs' ))

reads = as.numeric (system (paste0 ('wc -l ', Sample, '.seqs | cut -d\' \' -f1' ), intern=T))

bin = reads / TotalThread

for (p in 1:TotalThread){

threadtxt=sprintf ('%06d', p)

SampleThread=paste0 (Sample, '.thread', threadtxt)

binstart= ceiling((p - 1) * bin) +1

binend = min (ceiling(p * bin), reads)

system (paste0('awk \'NR>=', binstart, '&&NR<=', binend,'\' ', Sample, '.seqs > split/', Sample, '.', threadtxt ,'.seqs'))

}

file.remove(paste0('split/', Sample, '.mutantcounts1'))

file.remove(paste0('split/', Sample, '.mutantcounts2'))

**agrep.R**

pThread=as.numeric ( Sys.getenv ('pThread') )

threadtxt=sprintf ('%06d', pThread)

samples = readLines ('samples.txt')

for (i in 1:length(samples)){

Sample=samples[i]

seqfile=paste0('split/', Sample, '.', threadtxt, '.seqs')

system (paste0('agrep -1 -D2 -I2 -c -e \'ACCGCGCCCGGCC(GTGTTTTCTTTGG)\' ',seqfile, ' >> split/',Sample, '.mutantcounts1'))

system (paste0('agrep -1 -D2 -I2 -c -e \'(CCAAAGAAAACAC)GGCCGGGCGCGGT\' ',seqfile, ' >> split/',Sample, '.mutantcounts2'))

system (paste0('agrep -1 -D2 -I2 -c -e \'GTTATCAGTATAT(GTGTTTTCTTTGG)\' ',seqfile, ' >> split/',Sample, '.wildcounts1'))

system (paste0('agrep -1 -D2 -I2 -c -e \'(CCAAAGAAAACAC)ATATACTGATAAC\' ',seqfile, ' >> split/',Sample, '.wildcounts2'))

}

**Fastqsplit.R**

samples = readLines ('samples.txt')

for (i in 1:length(samples)){

Sample=samples[i]

mutcount1=sum(as.numeric(readLines(paste0('split/',Sample, '.mutantcounts1'))))

mutcount2=sum(as.numeric(readLines(paste0('split/',Sample, '.mutantcounts2'))))

wildcount1=sum(as.numeric(readLines(paste0('split/',Sample, '.wildcounts1'))))

wildcount2=sum(as.numeric(readLines(paste0('split/',Sample, '.wildcounts2'))))

writeLines (paste0 ('pattern\tcounts\nACCGCGCCCGGCC(GTGTTTTCTTTGG)\t',mutcount1, '\n(CCAAAGAAAACAC)GGCCGGGCGCGGT\t', mutcount2, '\nGTTATCAGTATAT(GTGTTTTCTTTGG)\t', wildcount1, '\n(CCAAAGAAAACAC)ATATACTGATAAC\t', wildcount2), paste0(Sample, '.alugrep.results.tsv'))

}

mutcounttotal= mutcount1+ mutcount2

wildcounttotal= wildcount1+ wildcount2

VAF= mutcounttotal / (mutcounttotal+ wildcounttotal)

if (VAF < 0.1) writeLines ('No AluY insertion was found in exon 4 of RP1 at 8:55540494 position (hg19)', paste0(Sample, '.alugrep.final.results.txt'))

if (VAF >= 0.1 & VAF < 0.3) writeLines ('AluY insertion was suspected in exon 4 of RP1 at 8:55540494 position (hg19). Please recheck by visualizing AluY sequence at 8:55540494 position (hg19).', paste0(Sample, '.alugrep.final.results.txt'))

if (VAF >= 0.3) writeLines ('AluY insertion was detected in exon 4 of RP1 at 8:55540494 position (hg19).', paste0(Sample, '.alugrep.final.results.txt'))

**Table S1. Predictive pathogenicity scores and population frequency for *RP1* variant M1-M3, and *Alu*.**

| **Gene** | **Transcript** | **Nucleotide** | **Amino acid** | **CADD** | **FATHMM** | **gnomAD** | **Classification** | **Pathogenicity criteria** | **Novel variant** |
| --- | --- | --- | --- | --- | --- | --- | --- | --- | --- |
| *RP1* | NM_006269.1 | c.4582_4585del | p.(Ile1528Valfs*10) | 25.6 | 0.913 | 4/281542 | Pathogenic | PVS1, PM2, PM3, PP5 | Previously reported |
| *RP1* | NM_006269.1 | c.5797C>T | p.(Arg1933*) | 38 | 0.594 | 49/281934 | Not Applicable* | Not Applicable* | Previously reported |
| *RP1* | NM_006269.1 | c.4196del | p.(Cys1399Leufs*5) | 22.1 | 0.926 | 1/250622 | Pathogenic | PVS1, PM2, PM3, PP5 | Previously reported |
| *RP1* | NM_006269.1 | c.4052_4053ins328 | p.(Tyr1352Alafs*9) | 31 | 0.963 | Not found | Pathogenic | PVS1, PM2, PM3, PP5 | Previously reported |

Legends: Combined Annotation Dependent Depletion v1.6 (GRCh37) Phread values ≥15 are predicted pathogenic; Functional Analysis through Hidden Marknov Models (FATHMM) v2.3 or FATHMM-Indel was used for the scoring; Genome Aggregation Database (gnomAD) v.2.1.1 was used.

*Because the p.(Arg1933*) is almost polymorphic and shows quasi-Mendelian inheritance, it is difficult to apply it to ACMG classification.

ACMG classification of variants:

PVS1: null variant (nonsense, frameshift, canonical +- 2 splice sites, initiation codon, single or multiexon deletion) in a gene where LOF is a known mechanism of disease.

PM2: Absent from controls (or at extremely low frequency if recessive) in Exome Sequencing Project, 1000 Genomes Project, or Exome Aggregation Consortium.

PM3: For recessive disorders, detected in trans with a pathogenic variant.

PP5: Reputable source recently reports variant as pathogenic, but the evidence is not available to the laboratory to perform an independent evaluation.

**Table S2. Specificity and Sensitivity of *In Silico* grep search code to Detect the *RP1*-*Alu* insertion**

| Patient | Sample  genotype | Sequencing methods | Variant read count | Reference  read count | Variant allele frequency | Interpretation |
| --- | --- | --- | --- | --- | --- | --- |
| A.II-2 | Proband | WES | 22 | 56 | 0.282 | *Alu*Y insertion was suspected in exon 4 of *RP1* |
| B.II-1 | Proband | Panel | 213 | 707 | 0.232 | *Alu*Y insertion was suspected in exon 4 of *RP1* |
|  |  | WES | 11 | 35 | 0.239 | *Alu*Y insertion was suspected in exon 4 of *RP1* |
|  |  | WGS | 17 | 21 | 0.447 | *Alu*Y insertion was detected in exon 4 of *RP1* |
| C.II-2 | Proband | WES | 9 | 46 | 0.164 | *Alu*Y insertion was suspected in exon 4 of *RP1* |
| D.II-2 | Proband | WES | 36 | 58 | 0.383 | *Alu*Y insertion was detected in exon 4 of *RP1* |
|  |  | WGS | 13 | 21 | 0.382 | *Alu*Y insertion was detected in exon 4 of *RP1* |
| D.1-2 | Mother | WGS | 19 | 23 | 0.452 | *Alu*Y insertion was detected in exon 4 of *RP1* |
| E.II-1 | Proband | Panel | 247 | 848 | 0.226 | *Alu*Y insertion was suspected in exon 4 of *RP1* |

WES: whole exome sequencing; WGS: whole genome sequencing

**Table S3. *RP1*-*Alu* detection, Variant calling format parameters, total number of mobile elements, and runtime using MELT, Mobster, and SCRAMble algorithms.**

| Patient | Sequencing methods | Algorithm | Insertion | MEI family | TSD | No. of MEIs | Runtime (sec) |
| --- | --- | --- | --- | --- | --- | --- | --- |
| A.II-2 | WES | MELT | 8:55540494 | ALU | NF | 12 | 205 |
|  |  | Mobster | 8:55540494 | ALU | NF | 67 | 247 |
|  |  | SCRAMble | 8:55540494 | ALU | NF | 12 | 205 |
| B,II-1 | Targeted | MELT | NF | NF | NF | 1 | 73 |
|  |  | Mobster | 8:55540483 | ALU | Duplication | 8 | 142 |
|  |  | SCRAMble | 8:55540494 | ALU | AAAACAC | 18 | 135 |
|  | WES | MELT | 8:55540494 | ALU | NF | 9 | 141 |
|  |  | Mobster | 8:55540494 | ALU | NF | 11 | 278 |
|  |  | SCRAMble | 8:55540494 | ALU | NF | 10 | 140 |
|  | WGS | MELT | 8:55540494 | ALU | AAAGAAAACAC | 1344 | 5227 |
|  |  | Mobster | 8:55540483 | ALU | Duplication | 1306 | 3863 |
|  |  | SCRAMble | 8:55540494 | ALU | NF | 2437 | 4184 |
| C.II-2 | WES | MELT | 8:55540494 | ALU | NF | 23 | 217 |
|  |  | Mobster | NF | NF | NF | 59 | 245 |
|  |  | SCRAMble | NF | NF | NF | 13 | 208 |
| D.II-2 | WES | MELT | 8:55540494 | ALU | AAAGAAAACAC | 35 | 263 |
|  |  | Mobster | 8:55540494 | ALU | NF | 100 | 268 |
|  |  | SCRAMble | 8:55540494 | ALU | NF | 24 | 140 |
|  | WGS | MELT | 8:55540494 | ALU | AAAGAAAACAC | 1363 | 7804 |
|  |  | Mobster | 8:55540483 | ALU | Duplication | 1302 | 5046 |
|  |  | SCRAMble | 8:55540494 | ALU | NF | 2401 | 4267 |
| D.I-2 | WGS | MELT | 8:55540494 | ALU | AAAGAAAACAC | 1398 | 5460 |
|  |  | Mobster | 8:55540483 | ALU | Duplication | 1295 | 4238 |
|  |  | SCRAMble | 8:55540494 | ALU | AAAGAAAACAC | 2863 | 5712 |
| E.II-1 | Targeted | MELT | NF | NF | NF | 9 | 130 |
|  |  | Mobster | 8:55540483 | ALU | Duplication | 11 | 256 |
|  |  | SCRAMble | 8:55540494 | ALU | NF | 2 | 50 |

MEI: mobile element insertion; NF: not found; TSD: target site duplication; WES: whole exome sequencing; WGS: whole genome sequencing

**
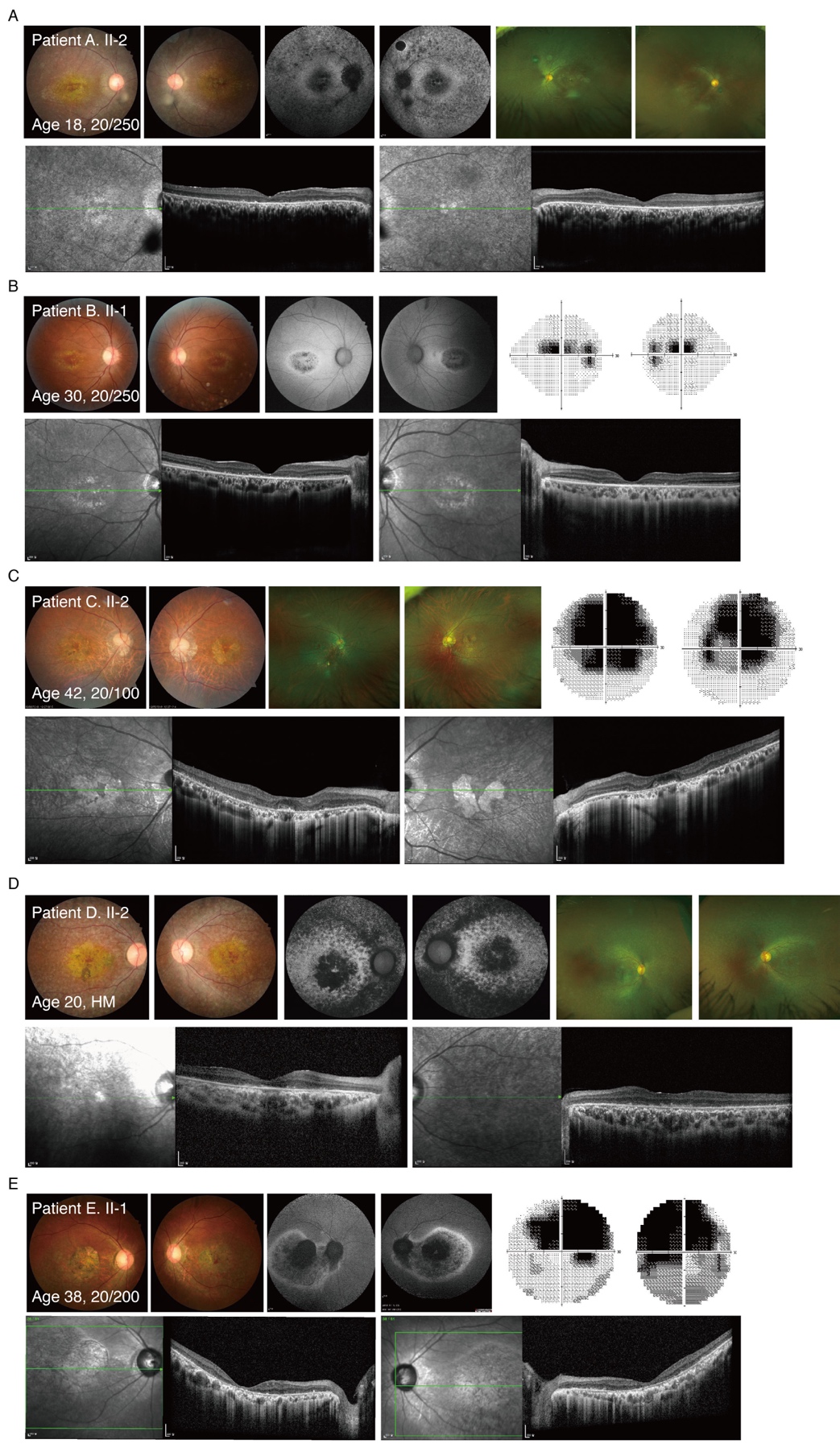
**

**Figure S1.** Detailed clinical information of 5 patients with *RP1* *Alu* insertions. Automated visualfield tests were not available in 2 patients (Patient A and D) due to nystagmus. Patients B, D, and E showed macular dystrophy without peripheral retinal degeneration. Automated visual field test showed dense central scotoma. Patient A and D had childhood onset nystagmus and fundus autofluorescence showed hypofluorescence at macular and major vascular arcade, which is consistent with cone rod dystrophy.

**
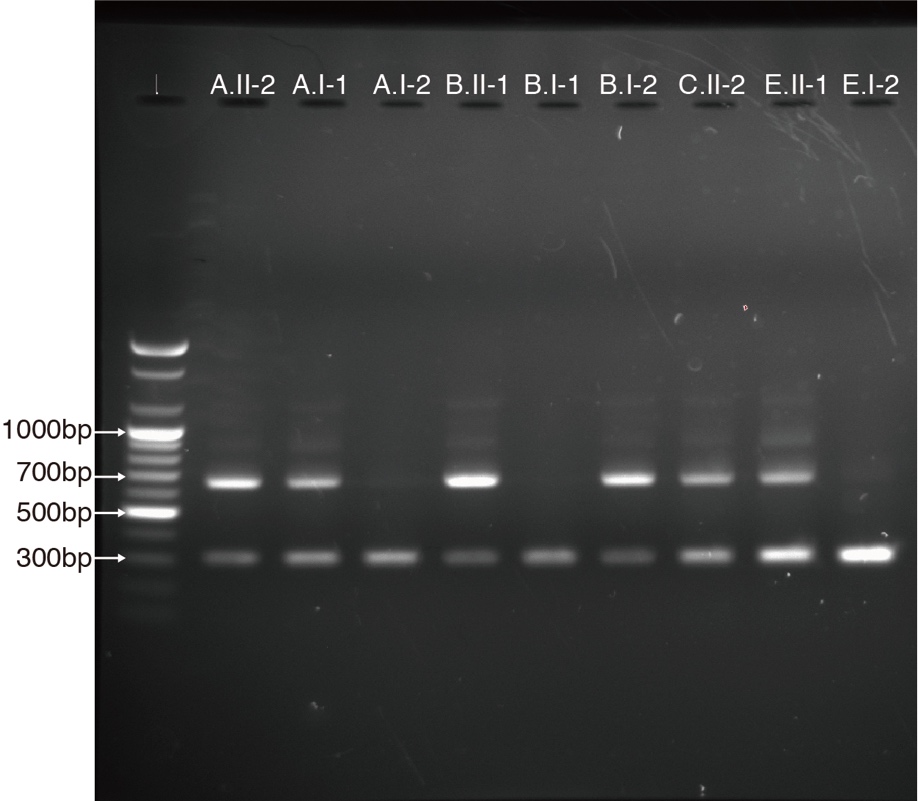
**

**Figure S2.** Confirmation of the *Alu* insertion by PCR and gel electrophoresis. DNA fragments containing the *Alu* insertion were distinguished from DNA fragments not containing the *Alu* insertion by size. The samples were derived from the same experiment and gels were processed in parallel.

**Forward**

TGAGACCTACGTTCCTGTCAATGTCTGCAATACCATTGACTTTTTAAACTCCAAAGAAAACACGGCCGGGCGCGGTGGCTCACGCCTGTAATCCCAGCACTTTGGGAGGCCGAGGCGGGCGGATCACGAGGTCAGGAGATCGAGACCATCCCGGCTAAAACGGTGAAACCCCGTCTCTACTAAAAATACAAAAAATTAGCCGGGCGTAGTGGCGGGCGCCTGTAGTCCCAGCTACTTGGGAGGCTGAGGCAGGAGAATGGCGTGAACCCGGGAGGCGGAGCTTGCAGTGAGCCGAGATTGCGCCACTGCACTCCAGCCTGGGCGACAGAGCGAGACTCCGTCTCAAAAAAAAAAAAAAAAAAAAAAAAAAAAAAAAAAAAAAAAAAAAAAAAAAAAAAAAA


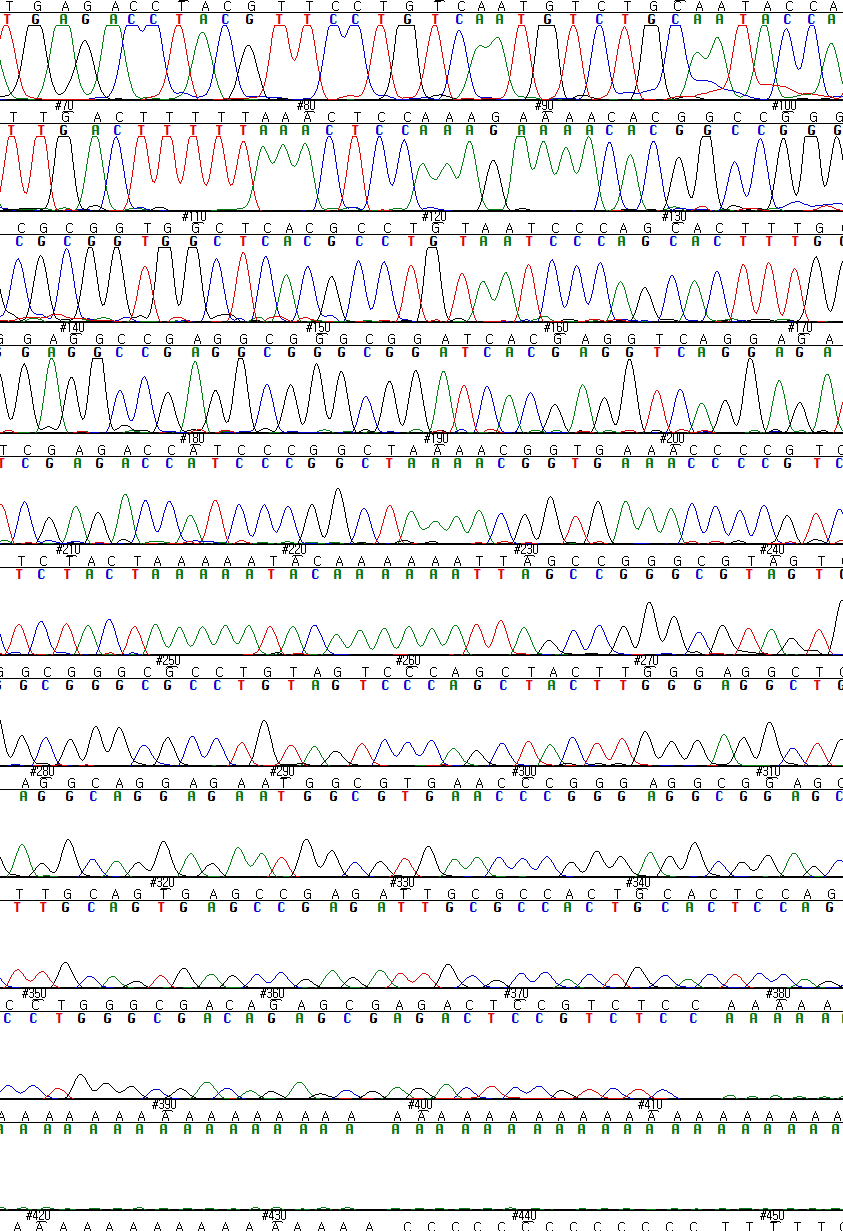


**Reverse**

AAAAAAAAAAAAAAAAAAAAAAAAAAAAAAAAAAAAAAAAAGAAAACACATATACTGATAACTTGGATTCAACTGAAGAGTTAGAAAGAGGTGATGACATTCAGAAAGATCTAAATATTT


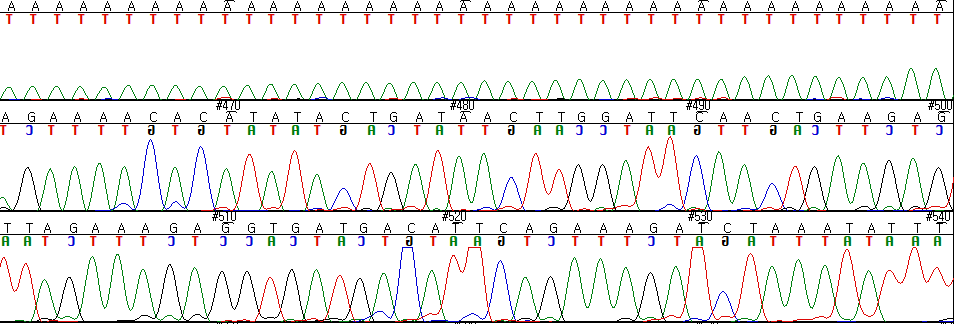


**Figure S3.** Sanger sequencing of *Alu* insertion. Each domain of the *Alu* is labelled by color: exon4 of *RP1*, target site duplication (TSD), *Alu*, polymorphic site of *Alu*Y and polyadenylation site.
